# cGMP production and analysis of BG505 SOSIP.664, an extensively glycosylated, trimeric HIV‐1 envelope glycoprotein vaccine candidate

**DOI:** 10.1002/bit.26498

**Published:** 2017-12-11

**Authors:** Antu K. Dey, Albert Cupo, Gabriel Ozorowski, Vaneet K. Sharma, Anna‐Janina Behrens, Eden P. Go, Thomas J. Ketas, Anila Yasmeen, Per J. Klasse, Eddy Sayeed, Heather Desaire, Max Crispin, Ian A. Wilson, Rogier W. Sanders, Thomas Hassell, Andrew B. Ward, John P. Moore

**Affiliations:** ^1^ International AIDS Vaccine Initiative New York New York; ^2^ Department of Microbiology and Immunology Weill Medical College of Cornell University New York New York; ^3^ Department of Integrative Structural and Computational Biology, International AIDS Vaccine Initiative (IAVI) Neutralizing Antibody Center and the Collaboration for AIDS Vaccine Discovery The Scripps Research Institute La Jolla California; ^4^ Department of Biochemistry, Oxford Glycobiology Institute University of Oxford Oxford UK; ^5^ Centre for Biological Sciences and Institute for Life Sciences University of Southampton Southampton UK; ^6^ Department of Chemistry The University of Kansas Lawrence Kansas; ^7^ Department of Medical Microbiology, Academic Medical Center University of Amsterdam Amsterdam The Netherlands

**Keywords:** affinity purification, cGMP, HIV‐1 vaccine, native‐like Env trimers, recombinant vaccine development, SOSIP

## Abstract

We describe the properties of BG505 SOSIP.664 HIV‐1 envelope glycoprotein trimers produced under current Good Manufacturing Practice (cGMP) conditions. These proteins are the first of a new generation of native‐like trimers that are the basis for many structure‐guided immunogen development programs aimed at devising how to induce broadly neutralizing antibodies (bNAbs) to HIV‐1 by vaccination. The successful translation of this prototype demonstrates the feasibility of producing similar immunogens on an appropriate scale and of an acceptable quality for Phase I experimental medicine clinical trials. BG505 SOSIP.664 trimers are extensively glycosylated, contain numerous disulfide bonds and require proteolytic cleavage, all properties that pose a substantial challenge to cGMP production. Our strategy involved creating a stable CHO cell line that was adapted to serum‐free culture conditions to produce envelope glycoproteins. The trimers were then purified by chromatographic methods using a 2G12 bNAb affinity column and size‐exclusion chromatography. The chosen procedures allowed any adventitious viruses to be cleared from the final product to the required extent of >12 log_10_. The final cGMP production run yielded 3.52 g (peptidic mass) of fully purified trimers (Drug Substance) from a 200 L bioreactor, a notable yield for such a complex glycoprotein. The purified trimers were fully native‐like as judged by negative‐stain electron microscopy, and were stable over a multi‐month period at room temperature or below and for at least 1 week at 50**°**C. Their antigenicity, disulfide bond patterns, and glycan composition were consistent with trimers produced on a research laboratory scale. The methods reported here should pave the way for the cGMP production of other native‐like Env glycoprotein trimers of various designs and genotypes.

## 1 INTRODUCTION

The quest for an effective vaccine to protect against human immunodeficiency virus type 1 (HIV‐1) infection continues. Various hypotheses about the nature of protective immunity are being explored using a variety of vaccine designs in animal models and human volunteers (Escolano et al., 2017; Haynes & Mascola, 2017; Korber, Hraber, Wagh, & Hahn, 2017; Safrit et al., 2016). One active area of research is to identify ways to induce broadly neutralizing antibodies (bNAbs) that target the envelope glycoprotein (Env) trimer on the surface of infectious HIV‐1 virions (Haynes & Mascola, 2017; Sanders & Moore, 2017; Stamatatos et al., 2017). Eliciting bNAbs is an attractive concept because of their ability to counter the extensive sequence diversity that is a critical aspect of the global HIV‐1 pandemic. However, despite many years of effort, no immunogen has yet been able to trigger this type of antibody response in non‐human primates or humans. Although Env proteins of various designs may, in principle, contribute to the elicitation of bNAbs, much effort is now being applied to making soluble, recombinant trimers that mimic the structure of the native form of virion‐associated Env (Sanders & Moore, 2017; Ward & Wilson, 2017). The first soluble trimer with appropriate in vitro properties was designated SOSIP.664, specifically one based on the BG505 clade A *env* gene (Sanders et al., 2015). The SOSIP.664 design includes the use of an engineered disulfide bond to covalently link and thereby stabilize the gp120 and gp41 ectodomain (gp41_ECTO_) subunits of the trimer, an I559P point substitution in gp41_ECTO_ that maintains the trimer in a pre‐fusion conformation, and a truncation in the C‐terminal region of gp41_ECTO_ that prevents unwanted aggregation (Sanders et al., 2013; Sanders & Moore, 2017). Co‐expression of the Furin protease maximizes cleavage of the gp160 precursor protein that is required for the resulting trimer to adopt a native‐like structure (Sanders & Moore, 2017; Ward & Wilson, 2017) and glycosylation (Behrens & Crispin, 2017; Pritchard et al., 2015). The resultant soluble trimers, of BG505 and other genotypes, have a native‐like structure as judged by multiple criteria including negative‐stain electron microscopy (NS‐EM), and they present epitopes for multiple bNAbs but few for non‐neutralizing antibodies (non‐NAbs) (Julien, Cupo, et al., 2013; Lyumkis et al., 2013; Pancera et al., 2014; Sanders et al., 2013; Sanders & Moore, 2017; Ward & Wilson, 2017). Several stability and/or antigenicity improvements to the prototypic BG505 SOSIP.664 trimer have since been described (Chuang et al., 2017; de Taeye et al., 2015; Ringe, Ozorowski, et al., 2017; Sanders & Moore, 2017; Torrents de la Pena et al., 2017).

When tested as immunogens in rabbits, guinea pigs and macaques, the original and improved versions of SOSIP.664 trimers of various genotypes induce a narrow‐specificity neutralizing antibody (NAb) response against the autologous Tier‐2 (i.e., neutralization‐resistant) virus, together with NAbs to Tier‐1 (i.e., neutralization‐sensitive) viruses (Cheng et al., 2015; Chuang et al., 2017; de Taeye et al., 2015; Klasse et al., 2016; Sanders & Moore, 2017; Sanders et al., 2015; Torrents de la Pena et al., 2017). The latter antibodies are predominantly directed against the V3 region of gp120, and their titers can be reduced by engineering variant trimers to suppress the immunogenicity of this region (Chuang et al., 2017; de Taeye et al., 2015; Ringe, Ozorowski, et al., 2017). Although low titers of NAbs against heterologous Tier‐2 viruses are occasionally elicited, SOSIP.664 and improved trimers have not induced bNAbs in the above species (Cheng et al., 2015; Chuang et al., 2017; de Taeye et al., 2015; Klasse et al., 2016; Sanders & Moore, 2017; Sanders et al., 2015; Torrents de la Pena et al., 2017). Nonetheless, native‐like trimers are key components of more sophisticated immunogen design and delivery programs aimed at eventually eliciting bNAbs (Medina‐Ramirez et al., 2017; Sanders & Moore, 2017; Stamatatos et al., 2017; Ward & Wilson, 2017).

A key question in the development of any vaccine is whether the vaccine candidate can be made in practical amounts at a reasonable cost and under current Good Manufacturing Practice (cGMP) conditions that are required for use in humans. HIV‐1 envelope glycoproteins of any design have not proven simple to produce as cGMP reagents, problems encountered including proteolytic damage (Wang et al., 2016; Yu, Fonseca, O'Rourke, & Berman, 2010) and aggregate formation (Finzi et al., 2010; Zambonelli et al., 2016) caused, in part, by the formation of aberrant disulfide bonds (Alam et al., 2013; Wieczorek et al., 2015; Zambonelli et al., 2016). Purification can also be problematic. These translational difficulties reflect the extensive glycosylation of HIV‐1 Env, with glycans comprising half the mass, and also the presence of nine intramolecular disulfide bonds in the gp120 subunit and another in gp41 (Behrens & Crispin, 2017; Go et al., 2017; Ward & Wilson, 2017). The processing state of the glycans has proven to be particularly sensitive to the precise quaternary structure of the Env complex (Behrens & Crispin, 2017; Pritchard et al., 2015).

Monomeric gp120 subunits and oligomeric gp140 HIV‐1 Env glycoproteins have been produced as cGMP‐grade immunogens, but the processes are usually not straightforward and often challenging and, furthermore, these proteins do not, in any case, mimic the native structure of the virus spike (Alam et al., 2013; Behrens & Crispin, 2017; Finzi et al., 2010; Wang et al., 2016; Ward & Wilson, 2017; Wieczorek et al., 2015; Zambonelli et al., 2016). The design of SOSIP.664 trimers is, seemingly, more complex than these earlier generation glycoproteins, so it was not clear whether they could be produced in useful amounts under cGMP conditions. Here, we show that cGMP‐quality BG505 SOSIP.664 trimers can indeed be made in multi‐gram quantities by adapting published, research laboratory methods involving a stable Chinese Hamster Ovary (CHO) cell line and a purification strategy based around a 2G12 bNAb‐affinity column (Chuang et al., 2017; Sanders et al., 2013). This purification strategy does not significantly skew the trimer glycosylation profile (Cao et al., 2017; Pritchard et al., 2015). The resulting trimers are pure, fully native‐like and highly stable for prolonged periods over a range of temperatures and incubation conditions.

## 2 MATERIALS AND METHODS

### 2.1 Production of stable CHO cell line

A CHO cell line expressing the BG505.SOSIP.664 Env protein, together with human Furin, was constructed under contract at Catalent Pharmaceutical Solutions (CPS) using their in‐house GPEx® retroviral expression system (Bleck, 2010, 2012). CPS designed the coding DNA sequences (CDS) and PCR primers needed to assemble the Env + Furin expression cassette into the retroviral vector, via unique restriction sites flanking the CDS. A Kozak sequence was included to increase protein expression efficiency and two stop codons were added to prevent translational read‐through events. The CDS for the full‐length human *furin* gene was excised from the CPS in‐house vector pCNS‐Human Furin‐WPRE GDD3276.0002. This *furin* CDS and the one for BG505 SOSIP.664 *env* were cloned by digestion of the PCR products with HindIII and XhoI, followed by ligation into the GPEx® plasmid pFCS‐new MCS‐WPRE‐SIN and digestion with the same enzymes to allow expression from the retroviral vector. Plasmids encoding BG505 SOSIP.664 and human Furin were named pFCS‐BG505SOSIP‐WPRE‐SIN and pFCS‐Human Furin‐WPRE‐SIN, respectively. Each plasmid CDS was verified by sequencing.

The two retroviral expression vectors were used to create the GPEx® CHO BG505 SOSIP.664 cell line by transduction of the GPEx® CHO parental line. Two transduction rounds with the Furin vector were followed by three with the BG505 SOSIP.664 vector. The pooled cell populations were expanded for cryopreservation after each round. The final cell pool was seeded into 96‐well cell culture plates to establish clonal cell lines by the limiting dilution cloning method. The clonal lines were screened for BG505 SOSIP.664 trimer production by PGT145‐ELISA (see SOM Methods). The top 20 clones, based on trimer content, were expanded and tested in duplicate 250 ml shake flask cultures. Their nutritional requirements for growth in a bioreactor were assessed in a standard fed‐batch culture study, and they were then cryopreserved.

### 2.2 Trimer harvest from CHO cells and purification

Culture supernatants containing BG505 SOSIP.664 gp140 proteins were harvested at day 15, or before viability dropped below 50%, and concentrated to ∼0.75 g/L (assessed by PGT145‐ELISA; SOM Methods) using a 300 kDa MWCO (Molecular Weight Cut‐Off) Polyethersulfone membrane. The concentrated supernatant was loaded onto a 2G12 monoclonal antibody (MAb)‐resin immunoaffinity column to bind the Env proteins for 5–6 cycles (i.e., the eluate was repeatedly passed through the column). The column was made using a cGMP stock of 2G12 Mab‐Toyopearl® resin purchased from Polymun Inc. (Klosterneuburg, Austria) (Joos et al., 2006; Trkola et al., 2008; Trkola et al., 1996). The loaded column was washed once with phosphate‐buffered saline (PBS), pH 7.4 and then with 20 mM Tris, 0.5 M NaCl, pH 8.0. Bound Env proteins were eluted using 50 mM Tris, 3 M MgCl_2_, pH 7.2. The eluted fraction was concentrated using a 300 kDa MWCO Regenerated Cellulose Membrane to a target concentration of ∼2 g/L and dia‐filtered six times into 20 mM Tris, 75 mM NaCl, pH 8.0. This gp140‐containing solution was treated with 0.5% Triton X–100 for 1 hr at room temperature as an initial virus‐inactivation step. The detergent was removed by batch absorption for 2 hr with Amberlite XAD‐2 (25.6 g per g of Triton X–100) followed by filtration. The clarified solution was loaded onto a ReadyToProcess MabSelect SuRe (Protein A) column to remove any 2G12 IgG that had leached from the immunoaffinity column. The flow‐through fraction containing gp140 proteins was next loaded onto a mixed‐mode anion exchange (MM‐AEX) Capto‐adhere column. The gp140 proteins in the flow‐through were passed through a Planova 20N nano‐filter as a viral clearance step, concentrated via a 100 kDa MWCO Regenerated Cellulose Membrane to a target concentration of ∼3 g/L, and loaded onto a Superdex 200 pg column, via 2% column volume injections. Here, the input material was divided into 6–10 aliquots for sequential size‐exclusion chromatography (SEC) cycles, with the relevant trimer‐fractions pooled. A final ultra‐filtration/dia‐filtration (UF/DF) step was performed using a 100 kDa MWCO Regenerated Cellulose membrane. The filtrate was concentrated to a trimer‐content of ∼3 g/L and dia‐filtered 10 times into Drug Substance/Drug Product (DS/DP) buffer, that is, 20 mM Tris, 100 mM NaCl, pH 7.5. This purified Bulk Drug Substance (BDS) was frozen and stored at −75 ± 10°C. To generate the Drug Product, the 3 g/L BDS stock solution was diluted to 2 g/L in DS/DP buffer, filtered through a 0.2 μm membrane, aliquoted into vials, frozen and stored at −75 ± 10°C.

### 2.3 Viral clearance study

Two “model” viruses were used following the recommendation of ICH Q5A guidelines (Viral Safety Evaluation of Biotechnology Products Derived from Cell Lines of Human or Animal Origin): xenotrophic murine leukemia virus (XMuLV), which is a large, 70–100 nm diameter, mammalian C‐type retrovirus endogenous to CHO cells, and mouse minute virus (MMV), a small, 18–24 nm diameter, non‐enveloped DNA parvovirus that is resistant to heat, lipid solvent and acid pH conditions. The virus‐clearance tests were carried out by deliberately adding (i.e., spiking) known amounts of a test virus to the different fractions obtained during the various production steps and demonstrating that it was successfully inactivated or removed by the subsequent steps. The tests therefore provide a measure of confidence that the same processes would also eliminate any unknown, unsuspected, harmful viruses. We monitored four purification process steps for their efficiencies at clearing the model viruses: (i) 2G12 MAb immuno‐affinity column; (ii) MM‐AEX column; (iii) detergent (Triton X–100) inactivation; (iv) nano‐filtration. The input material to be processed through each step was spiked with a known concentration of the test viruses and the column flow‐through or eluate, or the filtrate, was collected. The amounts of the viruses present in the input and processed materials were then quantified by qPCR and/or infectivity assays to determine the extent of virus inactivation or removal, with the reduction factor expressed on a log_10_ scale.

### 2.4 Analytical methods

Methods for analyzing purified trimers that are substantially based on published procedures are located in the Supplementary Online Material (SOM) section.

## 3 RESULTS

### 3.1 Strategy for BG505 SOSIP.664 trimer production and purification

We based our strategy for producing cGMP‐quality trimers on procedures that had worked efficiently in the research laboratory to make trimers on the 1–50 mg scale (Chung et al., 2014; Sanders et al., 2013; Sanders et al., 2015). Thus, we elected to make a cell line that co‐expressed the BG505 SOSIP.664 *env* and *furin* genes, and then adapted that line to growth in serum‐free medium. The secreted Env proteins were passed down a 2G12‐MAb immunoaffinity column, followed by the use of SEC to isolate the trimer fraction. Additional chromatographic procedures, detergent treatment, and an ultrafiltration step contributed to the cGMP‐required removal of adventitious, CHO cell line‐derived viruses. The production of native‐like trimers was monitored routinely by using an ELISA or a BLI‐assay that were both based on the selectivity of the PGT145 bNAb for a quaternary, trimer‐specific epitope (Chung et al., 2014; Lee et al., 2015; Sanders et al., 2013). The critical assay used to assess trimer quality was NS‐EM imaging (Khayat et al., 2013; Sanders et al., 2013, 2015). These methods are summarized in the SOM section.

### 3.2 Production of a stable CHO cell line and clone selection

The trimer‐producing cell line was produced under contract at CPS Inc. Under research laboratory conditions, the Weill Cornell Medical College (WCMC) group had produced both 293T and CHO cell lines expressing BG505 SOSIP.664 trimers, using the Flp‐In system purchased from Invitrogen and its own *env* + *furin* dual‐expression vector (Chung et al., 2014). CPS has its own proprietary retroviral transduction system, GPEx®, for cell line production. As both methods were potentially suitable, and as 293T and CHO cells were each viable options for cGMP production, we minimized risk by making both 293T and CHO cell lines using both the Flp‐in and GPEx® methods. Among the four cell line production attempts at CPS, the one that succeeded the quickest was the CHO cell line made using the GPEx® system; so we focused subsequent efforts on this line and did not pursue the other three options.

Production of a CHO research cell bank (RCB) at CPS involved multiple rounds of transduction of the GPEx® CHO parental cell line with a retrovector made from the two constructs developed to express the *furin* and BG505 SOSIP.664 *env* genes, followed by limiting dilution cloning (see Methods). The resulting clones were screened for native‐like trimer content using the PGT145‐based ELISA (SOM Methods). Thus, the clones were diluted to two viable cells per 200 µl of media and plated in two 96‐well cell culture plates, to establish sub‐clonal lines that originated from single cells. The top 24 sub‐clones, as judged by trimer content, were expanded and seeded into duplicate 250 ml shake flasks for a 14‐day fed‐batch culture. The quantity and quality of trimers in harvested culture supernatants were assessed by PGT145‐ELISA, BN‐PAGE and SDS–PAGE, the latter performed with and without reducing agent for evaluation of gp120‐gp41 cleavage. The average maximum level of trimer production of the sub‐clones, estimated by PGT145‐ELISA, was 106 mg/L compared to 82 mg/L for the pools. A second ELISA was used to detect other forms of Env in an assay based on the F105 non‐NAb against an epitope that is not exposed on native‐like BG505 SOSIP.664 trimers (Sanders et al., 2013; Yasmeen et al., 2014). Taken together, a strong signal in the PGT145‐ELISA combined with a low one in the F105‐ELISA is a good guide to the production of native‐like trimers. Various other properties of each sub‐clone were also measured, specifically retroviral vector components, bioburden, mycoplasma, and gene copy index. To verify the insert size, the BG505 SOSIP.664 *env* and the human *furin* sequences were checked to confirm each was present. The density of viable cells in each sub‐clonal line in the fed‐batch shake flasks was also measured. Each sub‐clonal line was cryopreserved for later viability testing. The above test results were used to select the four best performing sub‐clones (232‐4, 270‐4, 327‐9, 397‐3) for the RCB. Their key properties (estimated trimer yield, cell viability during growth and post‐thaw, gene copy index and insert verification) are summarized in Table 1.

**Table 1:** Properties of the four top CHO cell sub‐clones expressing BG505 SOSIP.664 gp140

Sub‐clone	Trimer production (mg/L)	Maximum cell density (viable cells/ml)	Viable cell density post‐thaw (cells/ml)	Cell viability post‐thaw (%)	Gene copy index
232‐4	229	29.0 x 10^5^	13.08 x 10^6^	86	4.48
270‐4	205	37.5 x 10^5^	14.94 x 10^6^	92	5.99
327‐9	118	74.7 x 10^5^	22.00 x 10^6^	98	5.54
397‐3	76	62.1 x 10^5^	15.50 x 10^6^	96	4.53

The sub‐clones were generated via the GPEx® method. The unpurified trimer concentrations in the supernatants were estimated using the PGT145‐ELISA. Maximum cell density was measured on day 10. The pre‐specified criteria for viable cell density post‐thaw and cell viability post thaw were >5 × 10^6^ cells/vial and >85%, respectively. All four sub‐clones passed pre‐specified criteria for Bioburden, Mycoplasma content and Retroviral vector component content and the appropriate insert size.

From the RCB, sub‐clone 270–4 was chosen for further process development and to establish a master cell bank (MCB). We first conducted an MCB stability study to assess whether this sub‐clone was suitably stable in respect of its viability, growth rate and secretion of native‐like (i.e., PGT145‐reactive) trimers during long‐term culture. To do so, the 270‐4 sub‐clone was thawed from the MCB and continuously cultured by serial passage for 42 generations in shake flasks using CD OptiCHO medium supplemented with 6 mM L‐glutamine. Cell samples were frozen at generations 0, 4, 8, 12, 17, 22, 28, 35, and 42 for later analysis. At that time, the cells from generations 0, 8, 17, 28, 35, and 42 were thawed and cultured, with the outcomes summarized in Table 2. Trimer expression, as measured using PGT145‐ELISA, varied from 85 ± 1 to 101 ± 20 mg/L among the six test samples, and the expression levels at the start (generation‐0) and end (generation‐42) were highly comparable. To assess the stability of the gene inserts, DNA was isolated from the same six samples and the copy number measured by real‐time PCR. It ranged from 5.55 to 5.94 gene copies per cell over the course of the experiment, which is a non‐significant variation based on overlapping standard deviations among the individual values. Viability was also comparable for all the samples (Table 2).

**Table 2:** Stability study on MCB sub‐clone 270–4

Generation	Trimer concentration (mean ± SD, mg/L)	Gene copy index (mean ± SD)	Cell viability (%)
0	85 ± 1	5.71 ± 0.33	96.4
8	119 ± 26	5.68 ± 0.27	93.1
17	117 ± 4	5.55 ± 0.29	93.7
28	121 ± 11	5.54 ± 0.37	97.8
35	165 ± 23	5.94 ± 0.21	97.7
42	101 ± 20	5.61 ± 0.22	99.2

Unpurified trimer concentrations in supernatants were estimated using the PGT145‐ELISA. SD, standard deviation. Gene copy numbers were measured by qPCR and % viability by microscopic counting of viable cells.

We concluded that sub‐clone 270‐4, which was the MCB for the BG505 SOSIP.664 trimer‐producing CHO cell line, is genetically stable, retains high cellular viability, and sustains its production of native‐like trimers over 42 generations. These properties drove the decision to proceed with the MCB for product development.

### 3.3 Upstream process development to produce Env proteins from the stable GPEx® CHO MCB

We chose to have the GPEx® CHO MCB (sub‐clone 270‐4) transferred to KBI Biopharma Inc. (KBI) for upstream process development and the production of Env proteins. To develop a cell culture process, we first carried out a feed‐screening study in shake flasks on a 1 L scale and several studies in a 3 L bioreactor to identify the optimal feeds, feeding strategy, and temperature shift conditions (e.g., whether a shift from 37°C to a lower temperature, such as 32°C or 34°C, could improve Env protein production). A seed train optimization study in shake flasks led to the choice of CD OptiCHO medium supplemented with 6 mM L‐glutamine and 0.25 g/L Cell Boost 4. The various small‐scale 1 L and 3 L culture studies established the key process parameters for larger scale production runs. The upstream and downstream processes were then evaluated and “locked” by performing a 50 L Confirmation Run, using a single vial from the MCB. After the successful completion of this run, the scalability of the process was evaluated by performing a 200 L Demonstration Run involving the use of a MCB‐derived seed train in an XDR 200 L single‐use bioreactor (SUB) (Fig. S1, Table S1). The goal was to assess whether the optimized process could eventually be applied to a cGMP Run on a 200 L scale. The same media and supplements were used for the Wave (50 L) and production bioreactors (200 L), except that the anti‐foaming agent 0.1% Poloxamer 188 was added to the bioreactor. The Demonstration Run produced 184 L of clarified harvest supernatant containing ∼21 g of unpurified BG505 SOSIP.664 trimer (i.e., 114 mg/L), as estimated in the PGT145‐BLI assay (see SOM Methods).

### 3.4 Purification of native‐like BG505 SOSIP.664 trimers

**Figure 1:**
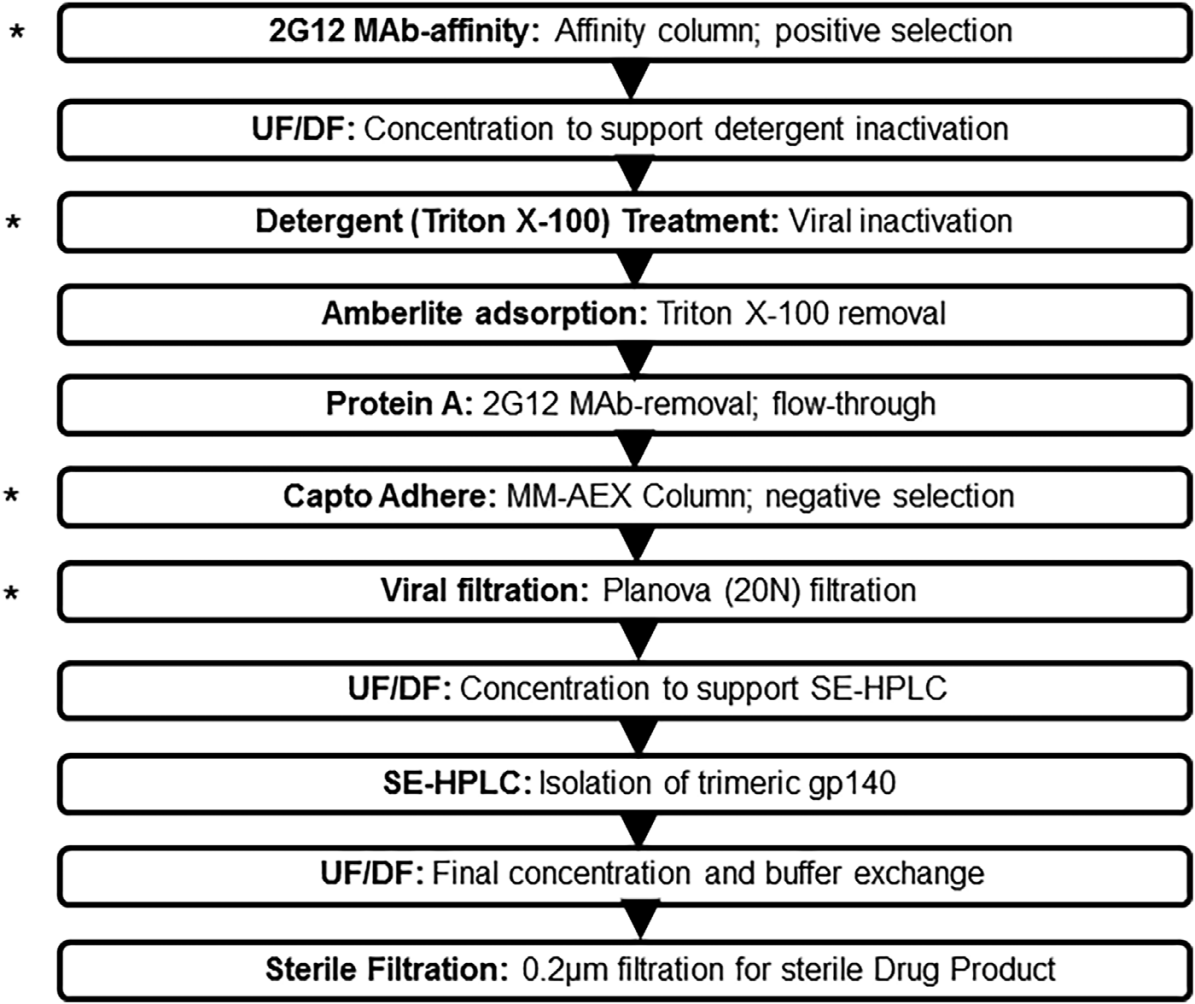
Overview of the process used to purify BG505 SOSIP.664 trimers. Schematic of the 10 steps involved in the purification of the trimer fraction from the initial gp140 protein harvest. The four dedicated viral clearance/inactivation steps are highlighted by an *. MM‐AEX, Mixed mode anion exchange

The trimer‐purification strategy was based around one that had been used successfully at the research laboratory level, that is, immuno‐affinity extraction of Env proteins from culture supernatants using a 2G12 bNAb‐Toyopearl® resin column, followed by SEC to purify the trimer fraction (Chung et al., 2014; Sanders et al., 2013; Sanders et al., 2015) (Figure 1). However, additional steps were incorporated into the cGMP process. A Protein A column (ReadyToProcess MabSelect SuRe) captured any 2G12 IgG that had leached from the affinity column, and an MM‐AEX Capto Adhere column to remove host‐cell protein and other process impurities before the SEC stage. The four column runs each contribute to the overall viral clearance/inactivation process, but two additional dedicated steps were also involved: Detergent (Triton X–100) inactivation and viral nano‐filtration (Planova 20N membrane).

Briefly, culture supernatant was harvested on day 15 or before cell viability dropped below 50%, concentrated and loaded onto a 2G12 MAb‐resin immunoaffinity column to allow capture of the BG505 SOSIP.664 gp140 proteins. After washing the column, the captured proteins were eluted using a neutral‐buffered solution of 3 M MgCl_2_, and the eluted fraction was rapidly diluted seven‐fold in 20 mM Tris, 75 mM NaCl, pH 8 and then further buffer‐exchanged and concentrated through UF/DF into the same buffer. The resulting solution was treated with Triton X–100 detergent as a virus‐inactivation step, the detergent was removed using Amberlite resin and the processed material passed down a Protein A column to trap any 2G12 MAb that may have leached from the immunoaffinity column. Non‐Env contaminants (e.g., host cell proteins) were then removed by passage down an MM‐AEX column. The gp140 proteins present in the eluate were then passed through the Planova nanofiltration system, another virus‐removal step, concentrated and then fractionated on a SEC column to isolate the trimers. The pooled trimer fractions were then filtered once more, this time in a UF/DF system, concentrated to ∼3 g/L and buffer‐exchanged via DF into the DS/DP buffer.

**Figure 2:**
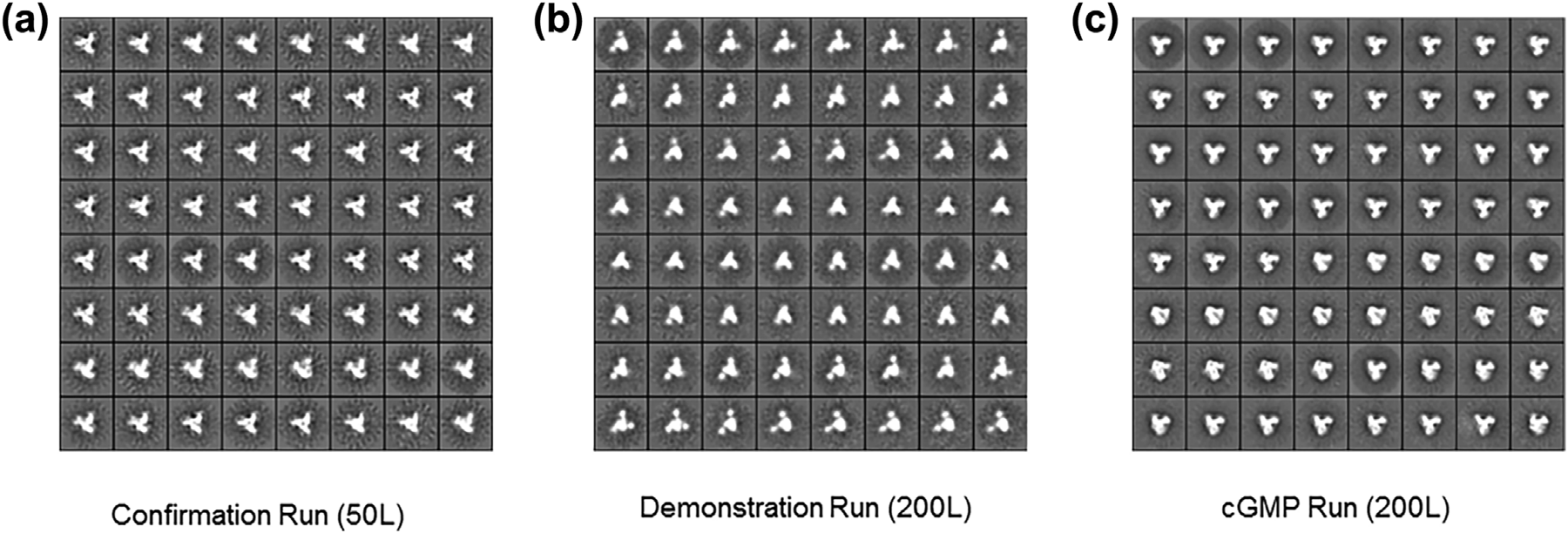
NS‐EM images of BG505 SOSIP.664 trimer Drug Substance products from small (50 L) and large (200 L) scale production runs. (a) 50 L Confirmation Run; (b) 200 L Demonstration Run; (c) 200 L cGMP Run. All three runs yielded trimers that were 100% native‐like

The robustness and effectiveness of the purification process was assessed by analyzing the Drug Substance products derived from the 50 L Confirmation Run, the 200 L Demonstration Run and the final 200 L cGMP Run (Table 3). In each case, the outcome was that >95% of the purified Env proteins were trimeric as determined by SEC, and >90% were PGT145‐reactive trimers as determined by PGT145‐BLI (Table 3). The purity of the trimers (i.e., the absence of other protein contaminants) was ≥90%, as determined by RP‐HPLC. We also verified that all process‐related contaminants (host cell protein, DNA, 2G12, Protein A) were present at levels that were either very low or below the Limit of Quantitation (LOQ) (Table 3). NS‐EM imaging showed that 100% of the trimers from all three of the production runs were in native‐like conformations (Figure 2). Thus, we purified 3.52 g of native‐like BG505 SOSIP.664 trimers using the chosen procedures, of which 1.8 g were used for filling the Drug Product vials. The rest was used for Drug Substance stability testing, stored as “retains” in satellite containers (for future testing, if required) or assigned to bio‐burden and other release testing studies.

**Table 3:** Comparison of small (50 L) and large (200 L) scale production runs used to produce the BG505 SOSIP.664 trimer Drug Substance

Attributes	Confirmation run (50 L)	Demonstration run (200 L)	cGMP run (200 L)
Concentration	13.5 mg/ml	2.8 mg/ml	3.0 mg/ml
Identity	Positive	Positive	Positive
Percent trimer (PGT145 BLI)	ND	121%	92%
Percent trimer (SEC)	100%	96.4%	96%
Purity (RP‐HPLC)	94.9%	90%	93.4%
Percent cleavage	Comparable to reference	Comparable to reference	Comparable to reference
Residual host cell protein	5 ppm	6 ppm	3 ng/mg
Residual DNA	<LOQ (15 ppb)	<LOQ (10 ppb)	<LOQ (2 pg/mg)
Residual 2G12 MAb	<LOQ (0.6 ppm)	<LOQ (8 ppm)	<17 ng/mg
Residual Protein A	<LOQ (0.05 ppm)	<LOQ (0.2 ppm)	<0.3 ng/mg
Endotoxin	ND	0.0398nEU/ml	0.02 EU/ml
Bioburden	ND	0 CFU/10mL	0 CFU/10ml
pH	7.5	7.5	7.5
Appearance	Clear, colorless solution	Clear, colorless solution	Clear, colorless solution

Attributes of the Drug Substance are listed together with the outcomes of the 50 L Confirmation and 200 L Demonstration runs (both non‐cGMP), and the 200 L cGMP run. ND, not determined; LOQ, limit of quantitation; EU, endotoxin units; CFU, colony forming units.

### 3.5 Viral clearance/inactivation

There is an inherent risk that any biotechnology product derived from a cell line could be contaminated by adventitious viruses present in the cell line itself (here, the CHO cells) or accidentally introduced during the production process. Because of the true or perceived risk to humans, regulatory agencies such as the Food and Drug Administration (FDA) require the use of a testing program to assess the inactivation or removal of viruses and thereby demonstrate that the resulting product meets recommended safety guidelines.

There are three main approaches to controlling potential viral contamination: (i) Selecting cell lines and other raw materials, including cell culture components, proven to be free of contaminating viruses that may be infectious in humans; (ii) testing the product during the production process to verify that no virus contamination has occurred; (iii) assessing the capacity of the production (i.e., the purification) processes to chemically inactivate or physically remove infectious viruses. Accordingly, we used FDA‐recommended procedures to test the CHO cell substrate, including the MCB, and relevant raw materials for viral contaminants. Since no viruses, virus‐like particles, or retrovirus‐like particles, were found in the cell substrate or other raw materials, we followed regulatory guidelines and assessed the capacity of the production process to clear “spiked” infectious model viruses. The objective of this viral clearance study was to identify steps in the BG505 SOSIP.664 trimer purification process that are expected to be effective in inactivating/removing viruses. These steps were then challenged by the addition of known amounts of spiked viruses to allow us to quantify the extent of virus load reduction achieved in each individual step, and overall. This information was used to estimate the risk of virus contamination in the final dosage form of the trimer product.

The overall purification process successfully removed/inactivated the model adventitious viruses by >12 log_10_, which is the minimum reduction required by regulatory guidelines using theoretical calculations based on the likely clinical dose of the BG505 SOSIP.664 trimers. The two dedicated viral clearance/inactivation steps based on non‐ionic detergent (Triton X–100) and nano‐filtration (Planova 20N) contributed substantially to viral inactivation and removal, respectively (Figure 1). Thus, Triton X–100 treatment inactivated the spiked input XuMLV by 5.96 log_10_. Since Triton X–100 is known to not inactivate the non‐enveloped MMV, we did not assess its effect on this virus. Filtration via the Planova 20N filter successfully removed both XMuLV and MMV by >5 log_10_ (Table 4). The 2G12 MAb immuno‐affinity and MM‐AEX column steps also contributed to viral clearance. The 2G12 column was particularly successful at removing the large XMuLV viruses (>5 log_10_) and also helped with the smaller MMV, although to a much lesser extent (∼1 log_10_). The MM‐AEX column run was highly effective in both cases, achieving reductions of >4 log_10_ for XMuLV and >6 log_10_ for MMV (Table 4). Cumulatively, these various individual steps achieved the required 13 log_10_ viral load reductions and without compromising the quality of the resulting trimers (Figure 2, Tables 3 and 4).

**Table 4:** Viral clearance/inactivation achieved during the process for purifying BG505 SOSIP.664 trimers

	Log_10_ virus reduction	
Purification process steps	XMuLV	MMV
2G12 MAb immuno‐affinity column	≥5.08	1.07
Detergent (Triton X–100, 0.5%) Inactivation	5.96	N/A
Capto Adhere (MM‐AEX) column	3.35	≥6.26
Viral filtration (Planova 20N)	≥5.23	≥5.00
Total	**≥19.62**	**≥12.33**

The viral load reduction (recorded as log_10_ compared to baseline) of XMuLV and/or MMV achieved by the four viral clearance/inactivation steps that are marked by an * in the overall purification process in Figure 2. DO, dissolved oxygen.

### 3.6 Characterization of purified BG505 SOSIP.664 trimers

**Figure 3:**
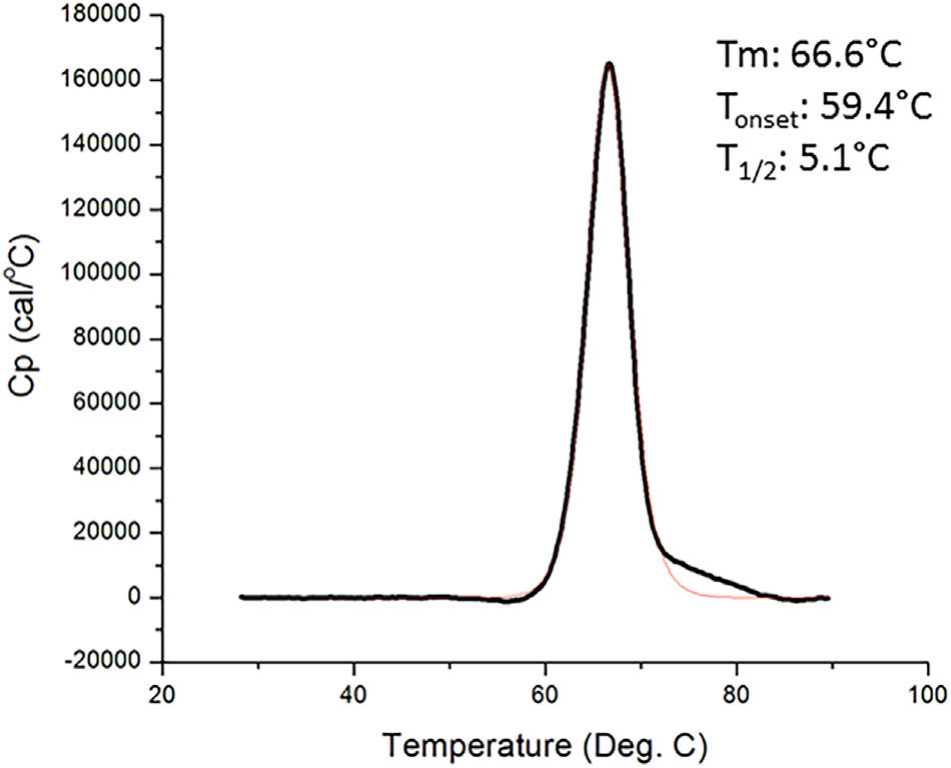
Properties of BG505 SOSIP.664 trimers purified from the Demonstration Run. (a) CD profile of BG505 SOSIP.664 trimer, (b) DSC profile of BG505 SOSIP.664 trimer showing a T_onset_ of 59.4°C and T_m_ of 66.6°C

Purified trimers from the Demonstration Run were studied in multiple assays to assess their quality and properties (Figures 2 and S2, Table 3). Env gp140 purity (i.e., absence of non‐Env contaminants), and trimer purity (i.e., absence of non‐trimeric forms of Env) properties were assessed by a variety of biochemical methods including SE‐HPLC, NS‐EM, PGT145‐BLI, and RP‐HPLC (see SOM Methods). Trimer purity was estimated to be ≥96% using SE‐HPLC. In addition, NS‐EM imaging showed that 100% of the visible Env proteins had native‐like trimer conformations (Figure 2). A comparison of reducing and non‐reducing SDS–PAGE gels confirmed that the purified trimers were fully cleaved (to the limit of detection) between the gp120 and gp140_ECTO_ subunits (Figure S2). This finding is consistent with the NS‐EM images. Hence, the Furin co‐expression method to maximize cleavage clearly works under scaled‐up production conditions. The same gels show that, as expected, the trimers were not proteolytically clipped in the gp120 V3 region or elsewhere (Figure S2). That particular problem commonly arises when monomeric gp120 proteins and some uncleaved gp140s are scaled up (Alam et al., 2013; Wang et al., 2016; Wieczorek et al., 2015; Zambonelli et al., 2016). Similarly, the trimers did not form aggregates, either via hydrophobic interactions or through the formation of aberrant inter‐molecular disulfide bonds, which is another frequently observed problem when uncleaved gp140s and some gp120s are produced on a large scale (Coutu & Finzi, 2015; Finzi et al., 2010; Go et al., 2015; Go, Hua, & Desaire, 2014; Go, Zhang, Menon, & Desaire, 2011; Ringe et al., 2015; Wang et al., 2016; Wieczorek et al., 2015; Zambonelli et al., 2016). DSC was used to analyze the thermal stability of the trimers. The spectrum showed a sharp thermal transition with an onset of unfolding at 59.4°C and a melting temperature of 66.6°C, consistent with an earlier report (Sanders et al., 2013) (Figure 3a).

**Figure 4:**
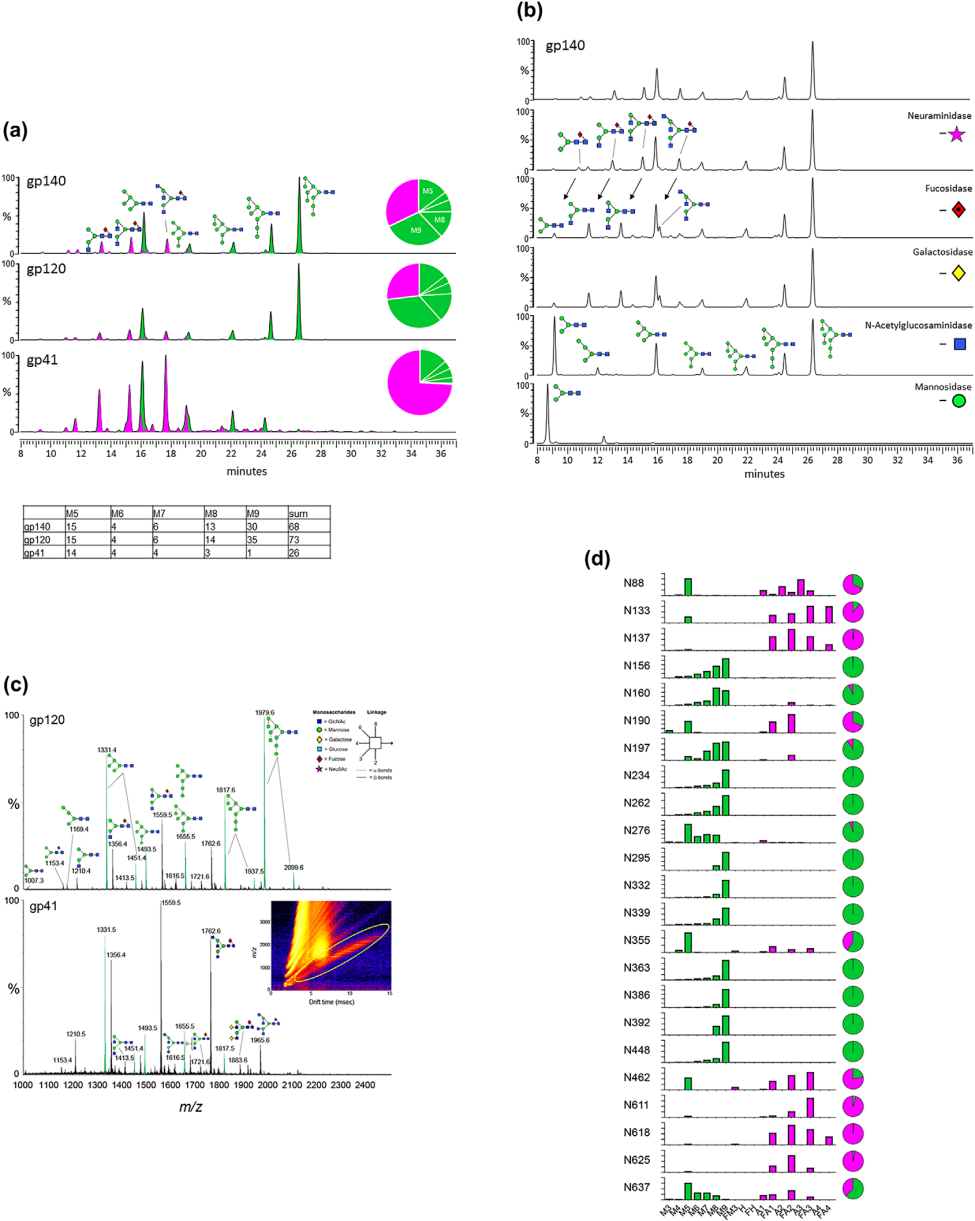
N‐linked glycosylation analysis. (a) Total glycan profiling of glycans enzymatically released from BG505 SOSIP.664 gp140 (200 L Demonstration Run), fluorescently labelled and analyzed by HILIC‐UPLC. Oligomannose‐type and hybrid glycans (green) were identified by their sensitivity to Endo H digestion. Peaks containing complex‐type glycans are colored in pink. The pie charts summarize the quantification of the peak areas. (b) Percentage quantification of the oligomannose‐type series Man5GlcNAc2 to Man9GlcNAc2 (M5 to M9) of the glycan profiles shown in a. (c) Glycan sequencing of glycan profile shown in a. Peaks were assigned by a sequential enzymatic digestion of 2‐AA labelled glycans with a panel of exoglycosidases, followed by HILIC‐UPLC analysis. The top panel shows the undigested glycan profile of BG505 SOSIP.664 trimers produced in CHO cells. The profiles below represent digestions with the following exoglycosidases: Neuraminidase from *Clostridium perfringens*, α‐L‐fucosidase from bovine kidney, β1, 4‐galactosidase from *Streptococcus pneumonia*, β‐N‐acetylglucosaminidase from *S. pneumonia* and Jack bean α‐mannosidase. (d) Quantitative site‐specific N‐glycosylation of 23 of the 28 sites on BG505 Env. The glycoprotein was digested with trypsin, chymotrypsin, pronase, and GluC, and then analyzed by LC‐ESI MS. Glycans are categorized as oligomannose series (M5 to M9), hybrids (H), and fucosylated hybrids (FH), and also by the number of branching antennae (A) of complex type glycans. An, number (n) of antennae; Gn, number (n) of galactose residues; F, presence of a core fucose (Behrens et al., 2016). The bar graphs represent the means of two analytical replicates, and the quantification of oligomannose‐type (green) and complex/hybrid‐type glycans (pink) on the individual sites is summarized in the pie charts

In a sialic acid content analysis, the molar ratio of Neu5Gc to BG505 SOSIP.664 was below the limit of quantitation (LOQ) of the assay (<0.011 mol/mol), while the corresponding molar ratio for Neu5Ac was 0.3 mol/mol. These findings are consistent with the UPLC analysis of enzymatically‐released glycans, which showed a minimal sialic acid content (Figure 4). The undetectable Neu5Gc content suggests that this component of the trimers would not be immunogenic in humans

Aberrant intra‐molecular disulfide bonds are commonly found in monomeric gp120 and uncleaved gp140 proteins (Alam et al., 2013; Coutu & Finzi, 2015; Finzi et al., 2010; Go et al., 2015, 2014, 2011; Ringe et al., 2015; Zambonelli et al., 2016). These events create non‐native sub‐populations on which relevant epitopes are not properly presented (Coutu & Finzi, 2015; Go et al., 2015; Ringe et al., 2015; Zambonelli et al., 2016). The disulfide bond profile of the purified trimers, as determined by mass spectrometry (MS), closely replicated the canonical profile of a research grade stock of this protein (Go et al., 2015). All of the native (canonical) disulfide bonded peptides were detected in high abundance (Table S3A). Two low‐abundant non‐canonical peptides were found at <5% abundance, that is, in trace amounts (Table S3B). These same peptides were also detected at <5% abundance when analyzing the research grade preparation of BG505 SOSIP.664 trimers (Go et al., 2015). Hence, the scaled‐up production process yielded a near‐homogenous population of trimers with the appropriate disulfide bond profile.

The free thiol group concentration of the trimers, as measured spectrophotometrically after treatment with Ellman's reagent, was below the LOQ of the assay (3.125 μM), and is therefore <0.7 mol of free thiol per mol of trimer. Free thiol groups were also sought in the MS data set but none was found. The estimated abundance of unpaired Cys in the trimers is <1%.

The glycan composition of the purified trimers showed that the gp120 component was dominated by the oligomannose‐type glycans, Man_9_GlcNc_2_ (M9), and Man_8_GlcNAc_2_ (M8), as found for the research grade stock of the same trimers, and that is considered to be a hallmark of a native‐like trimer conformation (Figure 4) (Behrens & Crispin, 2017; Behrens et al., 2016; Go et al., 2017; Pritchard et al., 2015). The site specific glycosylation also closely matched that reported for similarly analyzed research grade material (Figure 4d) (Behrens & Crispin, 2017; Behrens et al., 2016). Again, there are no indications that producing the BG505 SOSIP.664 trimers in much larger quantities in a stable CHO cell line decreases their quality.

**Figure 5:**
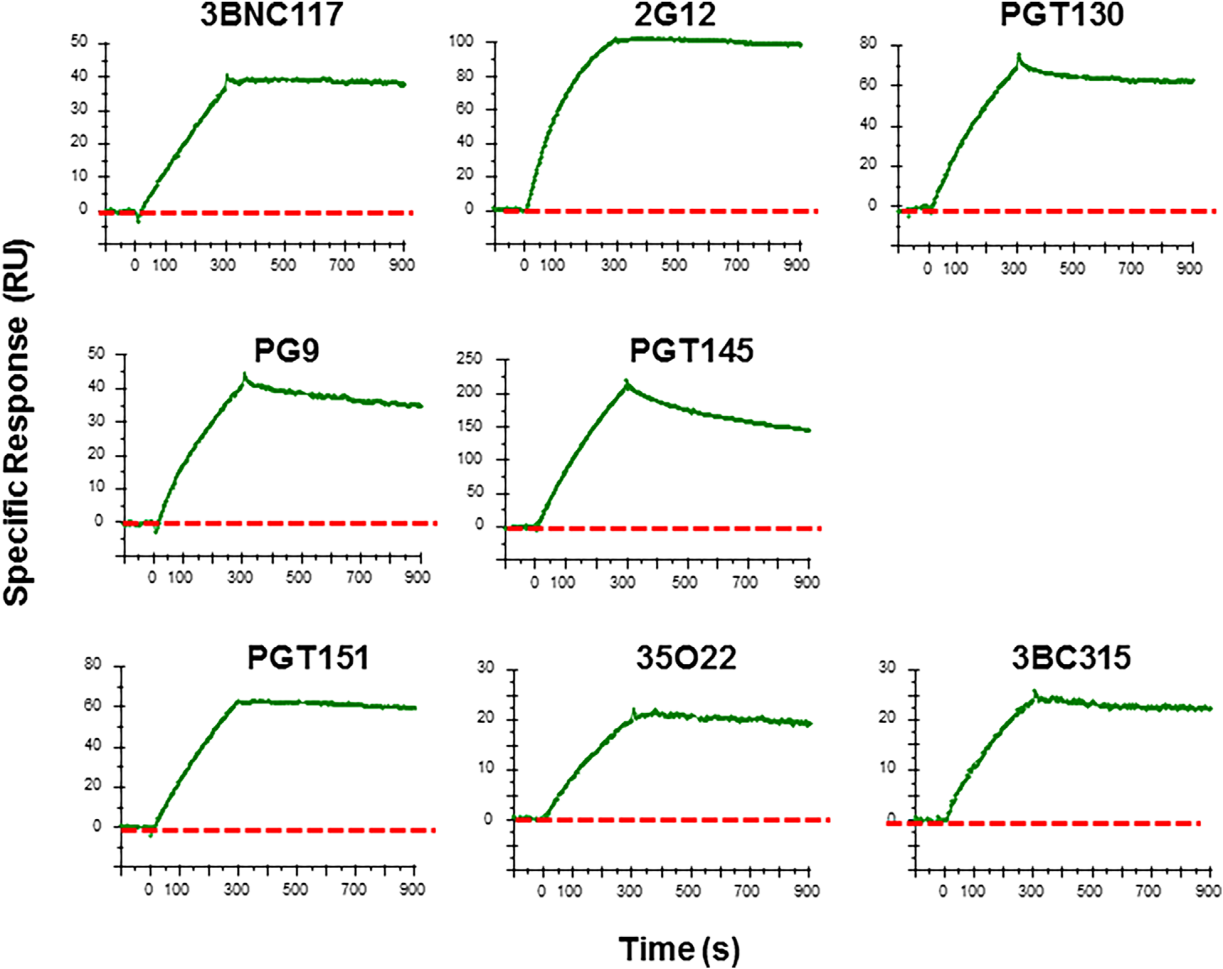
SPR analysis of bNAb binding to BG505 SOSIP.664 trimers. The binding of BG505 SOSIP.664 trimer in solution to the indicated immobilized bNAbs (3BNC117, 2G12, PGT130, PG9, PGT145, PGT151, 35022, and 3BC315) was analyzed by SPR. The green curves of the sensorgram show one of two replicate profiles of the specific response for each NAb (see SOM Methods) during 300 s of association and 600 s of dissociation. Note that the y‐axis scales are adjusted according to the maximum binding to each bNAb. The red dotted lines correspond to 0 response units (RU) on the y‐axis

In an SPR analysis, when the purified trimers were flowed over chip‐bound 3BNC117, 2G12, PGT130, PG9, PGT145, PGT151, 35O22, or 3BC315, the resulting binding profiles confirmed that the epitopes for all of these bNAbs were present (Figure 5).

### 3.7 Formulation development

Two central elements of vaccine formulation development are that key physicochemical and functional qualities of the immunogen remain stable, and that the immunogen can be formulated for safe administration to human volunteers. To determine the optimal storage condition at −75 ± 10°C, we performed a series of experiments in which we assessed the stability of purified BG505 SOSIP.664 trimers in a range of buffers, at different pH values, and with or without excipients. An initial Buffer Screening study tested the trimers at 2 mg/ml in three buffers, at −75 ± 10°C, at a range of pH values, and with and without 100 mM NaCl. The specific buffers were: 10 mM sodium citrate ± 100 mM NaCl at pH 5.0, 6.0, and 7.0; 20 mM sodium phosphate ± 100 mM NaCl at pH 6.5, 7.5, and 8.0; and 20 mM Tris ± 100 mM NaCl at pH 7.5, 8.0, and 8.5. When the formulated samples were analyzed using visual appearance, A280 (protein concentration), buffer pH, DSC (thermal stability), SEC (trimer size) and DLS (protein particle size homogeneity) endpoints (see SOM Methods), none of the buffer conditions was found to affect any measured parameter. A second Excipient Screening study was then conducted in which the trimers (2 mg/ml) were incubated for 1 week at 25 ± 2°C and 60 ± 5% relative humidity. The test buffers each contained 100 mM NaCl over a narrower pH range close to their pKa values, specifically 10 mM sodium citrate (pKa 6.4) at pH 6.5 and 7.0; 20 mM sodium phosphate (pKa 7.2) at pH 7.5 and 8.0; 20 mM Tris (pKa 8.1) at pH 7.5. Each buffer was tested with and without 150 mM L‐arginine or 250 mM sucrose to see whether either excipient conferred a stability increase (Table S4). As a control, additional trimer samples were incubated in the same buffers at 5 ± 3°C, with no added excipient. Based on the above endpoints, we observed no change to the properties of the trimers during the 1‐week incubation, irrespective of whether either arginine or sucrose was added as a potentially stabilizing excipient.

We then conducted an Accelerated Stability study in which a PGT145‐BLI endpoint was added to identify any changes in trimer conformation during prolonged storage. Trimers (2 mg/ml) were incubated for up to 4 weeks in 20 mM Tris, 100 mM NaCl at pH 7.5 with or without 250 mM sucrose at 5 ± 3°C, −20 ± 5°C, or −75 ± 10°C, and then assessed using the above endpoints of visual appearance, A280, pH, SEC, DLS, and PGT145‐BLI. No changes to the trimers were observed after the 4‐week incubation with no identifiable benefit of adding sucrose. In the final bulk freeze‐thaw study, the trimers were formulated at a higher concentration of 10 mg/ml in 20 mM Tris, 100 mM NaCl, pH 7.5 with and without sucrose, and then processed through five freeze‐thaw cycles between −75 ± 10°C and room temperature. The five cycles had no detectable effect on any endpoint, irrespective of the presence of sucrose. Accordingly, DS/DP (20 mM Tris, 100 mM NaCl, pH 7.5) was selected as the final formulation buffer for BG505 SOSIP.664 trimers.

### 3.8 Forced degradation study

This study was intended to identify BG505 SOSIP.664 trimer degradation pathways and products (see SOM for details). The purified trimers were stable under all test conditions except when oxidized with 0.04% H_2_O_2_ or when exposed to a low pH (3.5) solution (Table 5).

**Table 5:** Forced degradation study

(A) Summary of chemical and physical degradation stresses applied to purified BG505 SOSIP.664 trimers	
Sample	Condition
General control	Stored at −75 ± 10°C
Agitation	Orbital shaking at 200‐300 rpm for 24 and 48 hr
Agitation control	Samples stored at room temperature for 24 and 48 hr, without agitation
Oxidation	Exposed to 0.04% H_2_O_2_ at 37ºC for 4 hr
Heat stress	Incubated at 50ºC for 1 week
Freeze and thaw	Three cycles of freezing at −75°C for at least 2 hr followed by thawing at room temperature
Acid hydrolysis	pH 3.6 at 40°C for 3 days
Base deamidation	pH 8.9 at 40°C for 3 days
Photostability	8.00 kilolux of cool white light for 150 hr followed by 10.00 W/square meter UV light for 20 hr
Photostability control	Sample double wrapped with aluminum foil incubated in photostability chamber under same conditions

(B) Outcome of the stress tests as evaluated by physical appearance, solution pH, protein content (A280), PGT145‐binding (BLI), RP‐HPLC, SE‐HPLC and NS‐EM							
Conditions	Appearance	pH	Protein (mg/ml)	PGT145 binding (%)	RP‐HPLC (% in main peak)	SE‐HPLC (% in trimer peak)	NS‐EM trimer (%)
General control	Clear, colorless	7.5	1.9	100	92.7	96.0	100
Agitation 24 hr	Clear, colorless	7.5	2.0	104	92.4	95.8	100
Agitation 24 hr control	Clear, colorless	7.5	2.0	85	92.9	96.1	100
Agitation 48 hr	Clear, colorless	7.5	1.9	99	92.4	96.2	100
Agitation 48 hr control	Clear, colorless	7.5	2.0	87	92.7	95.8	100
Oxidation	Clear, colorless	7.5	2.0	**22**	**5.5**	94.7	100
Heat stress	Clear, colorless	7.5	1.9	84	89.7	94.9	100
Freeze thaw cycles	Clear, colorless	7.5	2.0	83	92.7	95.2	>90
Acid hydrolysis	Clear, colorless	3.6	2.0	**14**	**79.9**	**33.6**	**0**
Base deamidation	Clear, colorless	8.9	1.9	71	90.5	96.6	100
Photostability	Clear, colorless	7.5	2.0	84	91.6	96.0	100
Photostability control	Clear, colorless	7.5	2.0	86	92.7	96.1	100

Data indicative of degradation events are highlighted in bold.

## 4 DISCUSSION

Here, we describe the successful production of multi‐gram amounts of cGMP‐quality, BG505 SOSIP.664 trimers from a stable CHO cell line. An initial 50 L (small‐scale) run was followed by a 200 L non‐cGMP Demonstration Run and finally by a 200 L cGMP run. In each of the runs, the quality of the purified trimers was at least as high as those produced on a laboratory scale, but yields from the optimized process were ∼2‐fold greater (Chung et al., 2014; Sanders et al., 2013). Taking into account that the scale‐up involves a 10‐step purification process, as opposed to a two‐step one, the cGMP process overall was demonstrably efficient. The resulting cGMP‐grade trimers are highly pure and fully native‐like when assessed by NS‐EM, have the high oligomannose glycan content that is now considered a hallmark of native Env proteins, are not proteolytically clipped, have the expected, canonical disulfide bond profile, and display conformationally sensitive bNAb epitopes. In all respects, the cGMP‐quality trimers closely resemble those produced in research laboratories on a much smaller scale. Clearly, the basic procedures developed in the research laboratory were capable of successful scale‐up without compromising the properties of the end product. The final cGMP production run yield of 3.52 g of purified trimer represents a 58.4% recovery in the overall downstream purification process. That recovery is significantly higher than for previously manufactured HIV‐1 Env clinical trial candidates, and may be attributable to the efficiency of the mAb‐based affinity capture step. However, due to cGMP testing and stability requirements, only 1.8 g were used to prepare Drug Product vials containing 0.55 ml of a 2‐mg/ml trimer stock (i.e., 1.1 mg per vial). Note that the amount of trimers recorded is based on the peptidic content. The actual mass, accounting for the glycans, is ∼1.7‐fold higher. The optimal dose of appropriately adjuvanted trimers for human use, based on experiences in rhesus macaques, is likely to be in the range 100–300 μg, with at least three doses required to elicit an autologous NAb response.

When we contemplated starting this translational project, we were far from sure that it could be completed successfully. Many large‐scale HIV‐1 Env protein production programs have not proceeded smoothly and we were aware that the seeming complexity of SOSIP trimers, combined with the necessity for Furin co‐expression, could present additional challenges. In addition, we intended to use a purification strategy based on a bNAb affinity column. To our knowledge, this technique had never previously been reported for large‐scale, cGMP production of an Env protein. In practice, we encountered no serious obstacles that caused substantive delays. Taken into account only the time for “wet work,” the period between the initiating cell line production and the purification of the cGMP‐grade trimers, was only 24 months, far shorter than we expected based on general experiences gleaned from the Env vaccine field (n.b., the time taken to put in place legal agreements, including for the transfer of the CHO cell line from one contractor to another, added more than 6–8 months to the overall duration of the project).

There are several reasons for this rapid progress. A considerable amount of research had already established key procedures, including demonstrating that fully cleaved and native‐like BG505 SOSIP.664 trimers could be produced from Env + Furin co‐expressing 293T or CHO cell lines (Chung et al., 2014); showing that a 2G12 affinity column was a highly efficient way to start the trimer purification process combined with the knowledge that a cGMP‐grade stock of this bNAb already existed (Joos et al., 2006; Sanders et al., 2013; Trkola et al., 2008; Trkola et al., 1996); and assessing which virus‐inactivation procedures might be feasible for use in a cGMP production process. It was also prudent to evaluate four different ways to make a cGMP‐quality stable cell line. In this project, the one chosen was a CHO cell line made via the Catalent in‐house GPEx® system. We note, however, that we have since made four different SOSIP trimer‐expressing CHO cell lines successfully in a cGMP suite at the WCMC, via the same Flp‐in system used to produce multiple lines under standard laboratory conditions (Chung et al., 2014; Pugach et al., 2015; Ringe et al., 2017).

The above points notwithstanding, we judge that the principal reason for the rapid progress of this project is that the BG505 SOSIP.664 trimer was much simpler to produce on a relevant scale than earlier forms of Env, not harder. Because of how they fold into a compact, native‐like structure, SOSIP trimers are not vulnerable to proteolytic clipping (and any V3‐clipping for more sensitive clade B trimers can be eliminated by a point substitution) (Pugach et al., 2015). They are also not subject to the disulfide bond scrambling that creates improperly folded proteins, including disulfide‐linked aggregates (Go et al., 2015). The absence of surface exposed hydrophobic patches also reduces or even eliminates aggregate formation (Khayat et al., 2013; Klasse et al., 2013). All of these problems arise with gp120 monomers and uncleaved gp140s during production on a laboratory scale or greater (Alam et al., 2013; Coutu & Finzi, 2015; Finzi et al., 2010; Go et al., 2015; Go et al., 2014; Go et al., 2011; Ringe et al., 2015; Wang et al., 2016; Wieczorek et al., 2015; Yu et al., 2010; Zambonelli et al., 2016). The Forced Degradation study confirmed that the purified BG505 SOSIP.664 trimers were highly stable for prolonged periods, even at elevated temperatures. Only exposure to an acidic pH of 3.5 caused the trimers to disintegrate catastrophically into monomers and dimers, as judged by several techniques including NS‐EM, PGT145‐BLI, and SE‐HPLC. Oxidation caused by H_2_O_2_ damaged the trimers in a way that affected the PGT145 epitope and affected how the (oxidized) trimers migrated on RP‐HPLC. These various methods can, therefore, be used to monitor the stability of purified trimers during stability studies, in general.

The success of this translational project provides a template for ongoing or future projects aimed at producing large amounts of native‐like trimers of follow‐on designs. The procedures established here should be adaptable for many other trimers, provided that they are well designed and provably stable in laboratory‐based studies. One variable may be the identity of the bNAb chosen for purification. BG505 and several other genotypes of SOSIP trimer can be successfully purified using 2G12, but some cannot due to contamination by a subset of non‐native pseudo‐trimers that are not removed by an SEC column (Ringe et al., 2015). Other chromatographic procedures may be helpful here (Verkerke et al., 2016). However, our preferred option is to use a conformationally selective bNAb such as PGT145 to positively select only the native‐like trimer population (de Taeye et al., 2015; Pugach et al., 2015; Ringe et al., 2015). Anticipating that such a method might be useful, we suggested that PG145 should be produced as a cGMP‐grade reagent for trimer affinity purification. Programs to accomplish this goal are now under way in the USA and Europe. Several other bNAbs that are now being produced as cGMP‐grade reagents for clinical trials of passive antibody therapies in HIV‐1 infected people could also be evaluated for trimer purification (Escolano et al., 2017; Pancera et al., 2014).

Animal model research may eventually identify a moderate number of SOSIP trimers that, if used in combination, simultaneously or more likely sequentially, could induce bNAbs in humans. If so, we have now established that there is a reasonable probability that they could be produced under cGMP conditions in appropriate quantities without compromising their quality.

## CONFLICTS OF INTEREST

JPM, RWS, IAW, and ABW are listed on a patent filed by IAVI regarding BG505 SOSIP.664 trimers.

## Supporting information

Additional Supporting Information may be found online in the supporting information tab for this article.

**Figure S1**. Cell culture (i.e., upstream) process for BG505 SOSIP.664 gp140 production using GPEx® CHO stable cell clone 270‐4
**Figure S2**. Polyacrylamide gel electrophoretic analysis of BG505 SOSIP.664 trimers (A) BN‐PAGE
**Figure S3**. Oxidation of BG505 SOSIP.664 trimers
**Figure S4**. Acid treatment of BG505 SOSIP.664 trimers

**Table S1**. Process parameters used to produce BG505 SOSIP.664 trimers during theDemonstration Run and the cGMP Run

**Table S2**. Table S2. Amino acid analysis

**Table S3**. Table S3. Disulfide bonds present in BG505 SOSIP.664 trimers

**Table S4**. Excipient screening study
