# Supplementary figures and images for: cGMP production and analysis of BG505 SOSIP.664, an extensively glycosylated, trimeric HIV‐1 envelope glycoprotein vaccine candidate
Source: Biotechnol Bioeng. 2017 Dec 11;115(4):885–99. doi: 10.1002/bit.26498 (PMC5852640; doi:10.1002/bit.26498)

**Figure S1.**

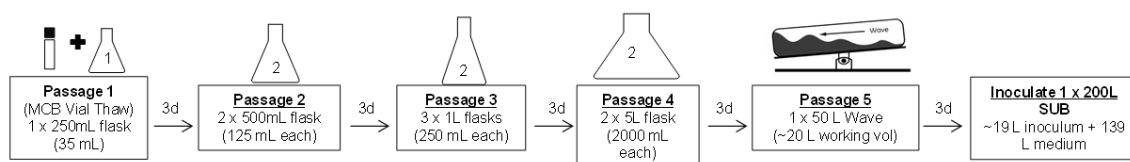

**Figure S2.**

**(A) BN-PAGE**

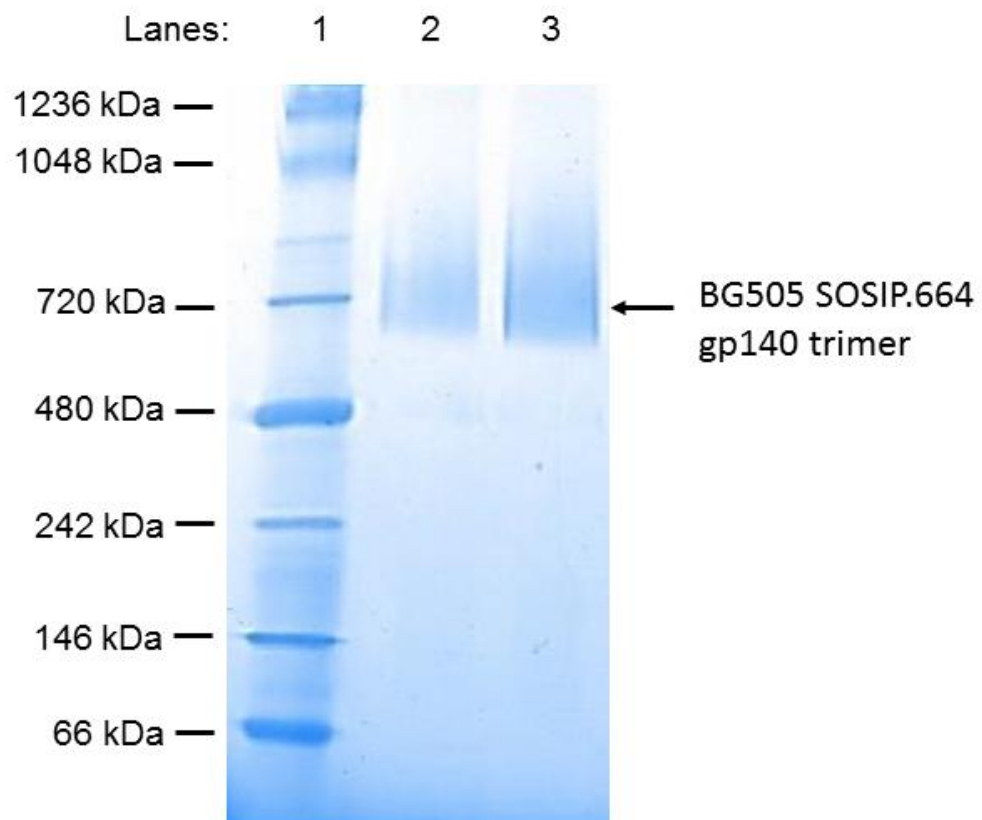

**Figure S2.**

**(B) SDS-PAGE**

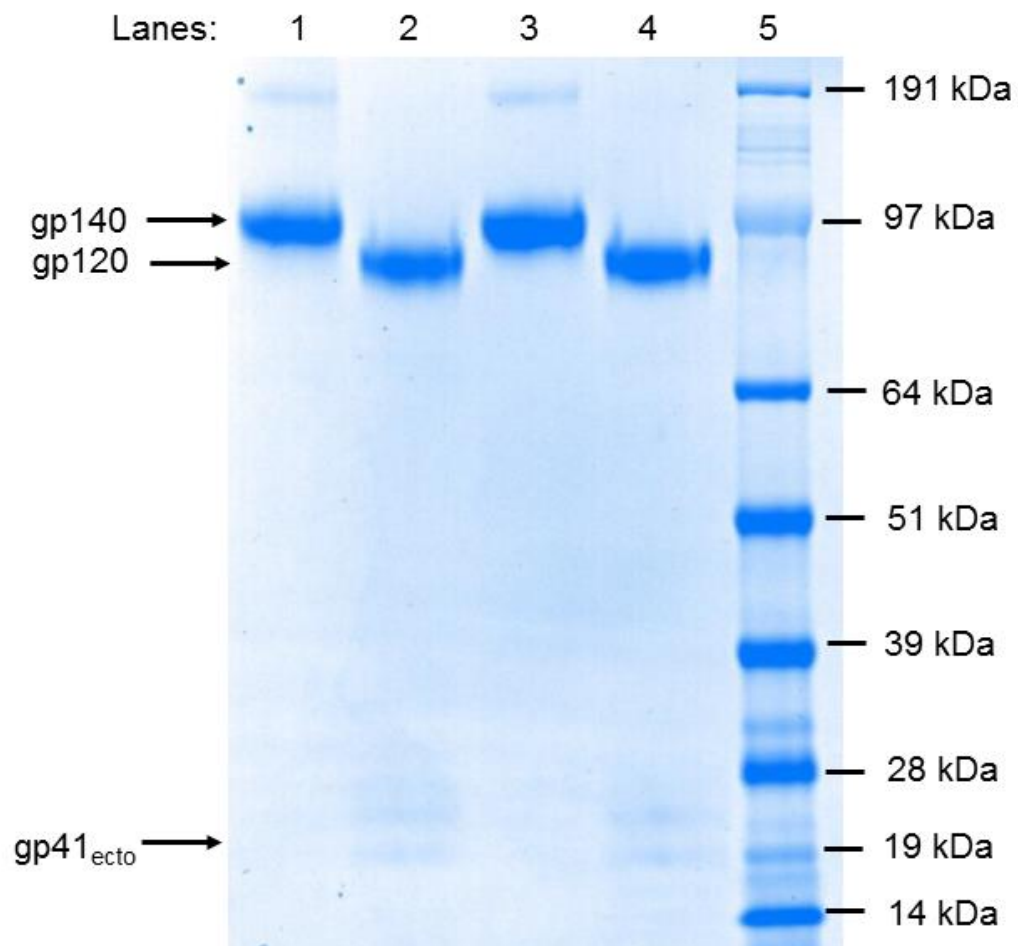

**Figure S3.**

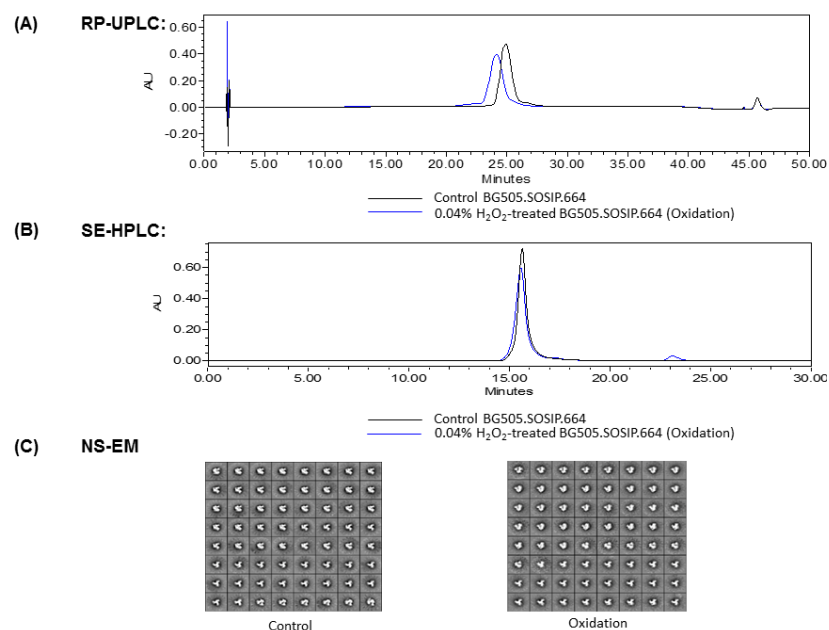

**Figure S4.**

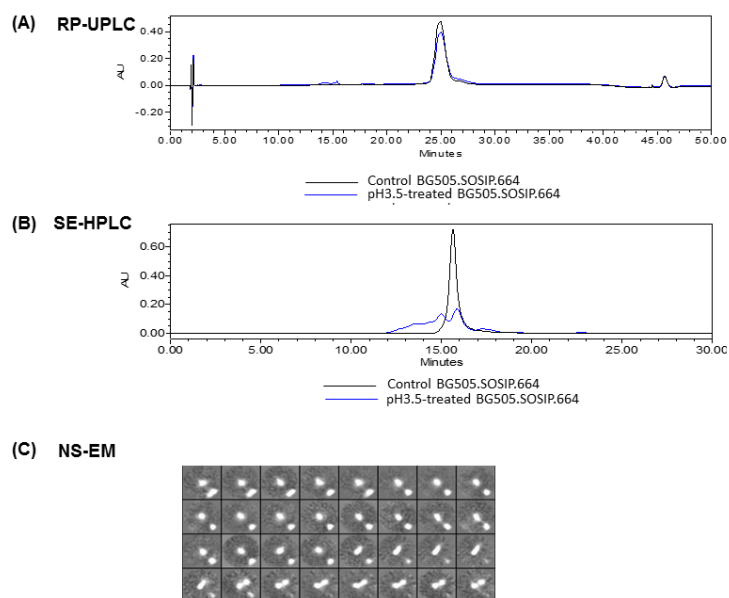

Supplement: Supplementary file 1 — Figure S1. Cell culture (i.e., upstream) process for BG505 SOSIP.664 gp140 production using GPEx® CHO stable cell clone 270‐4 Figure S2. Polyacrylamide gel electrophoretic analysis of BG505 SOSIP.664 trimers (A) BN‐PAGE Figure S3. Oxidation of BG505 SOSIP.664 trimers Figure S4. Acid treatment of BG505 SOSIP.664 trimers [file BIT-115-885-s001.pdf]

**Table S3.**

**(A)**


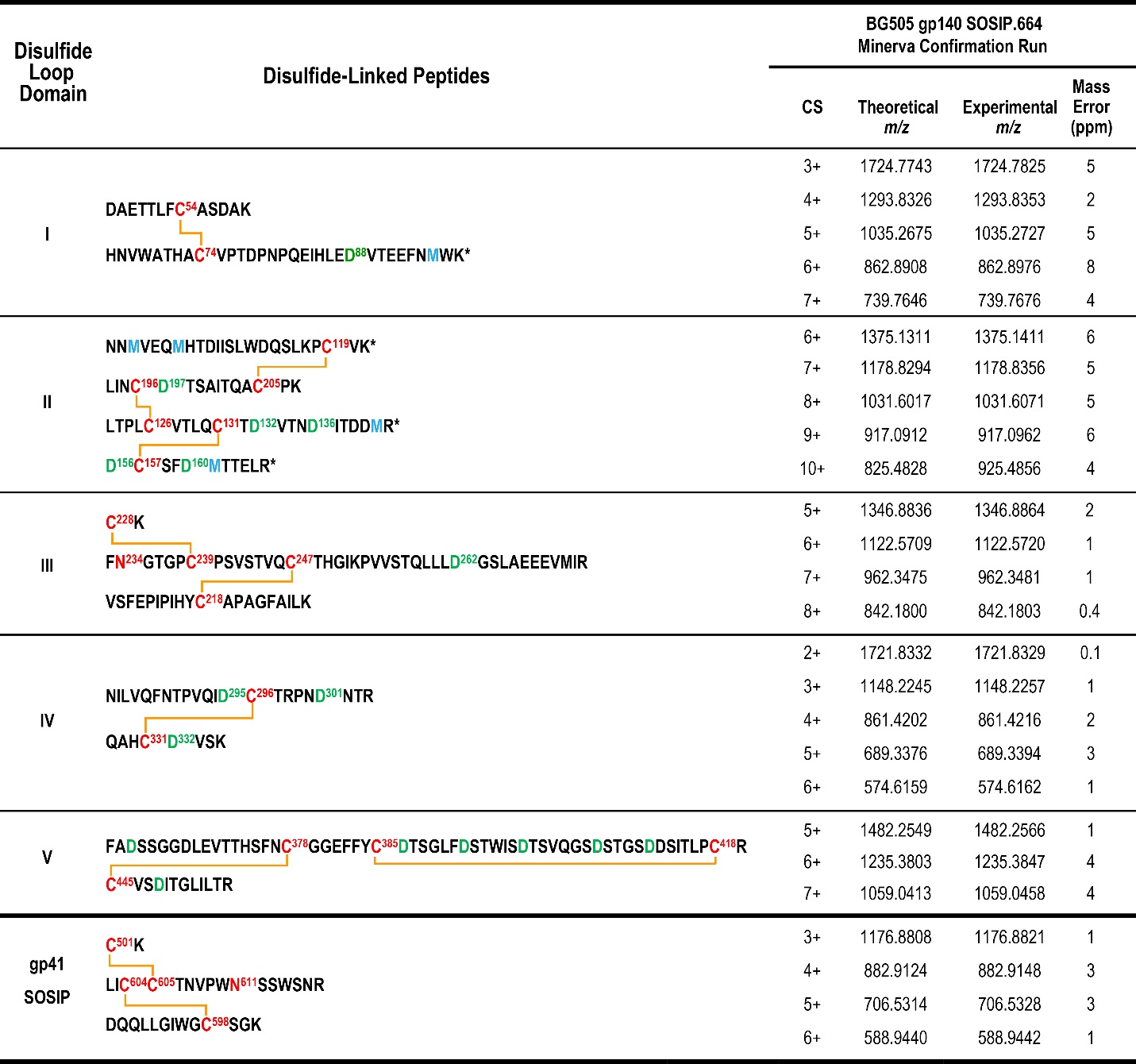


**(B)**

**
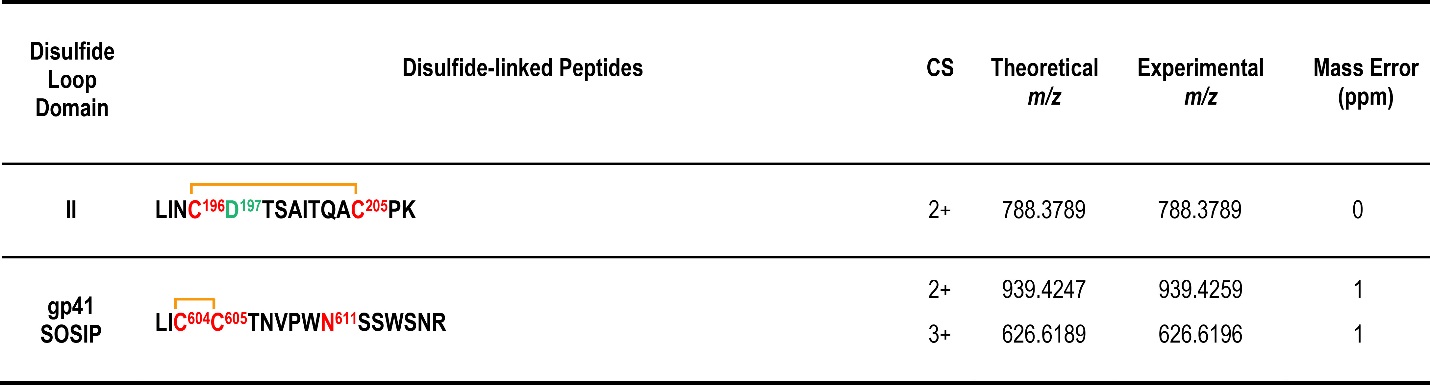
**

Supplement: Supplementary file 4 — Table S3. Table S3. Disulfide bonds present in BG505 SOSIP.664 trimers [file BIT-115-885-s004.docx]
