# Supplementary material for: cGMP production and analysis of BG505 SOSIP.664, an extensively glycosylated, trimeric HIV‐1 envelope glycoprotein vaccine candidate
Source: Biotechnol Bioeng. 2017 Dec 11;115(4):885–99. doi: 10.1002/bit.26498 (PMC5852640; doi:10.1002/bit.26498)
Supplement: Supplementary file 2 — Table S1. Process parameters used to produce BG505 SOSIP.664 trimers during theDemonstration Run and the cGMP Run [file BIT-115-885-s002.docx]

**Table S1.**

**(A)**

| **50 L Wave Bioreactor** | | | | | |
| --- | --- | --- | --- | --- | --- |
| **Target seeding density** | 0.30 x 10^6^ cells/mL | | | | |
| **Initial medium volume** | 18.5 L | | | | |
| **Temperature set point** | 37.0°C | | | | |
| **Rock rate** | Day | 0 | 1 | 2 | 3 |
|  | rpm | 18 | 20 | 22 | 22 |
| **Rock angle** | 8° | | | | |

**(B)**

| **200 L XDR Single-Use Bioreactor** | |
| --- | --- |
| **Temperature set point** | 37°C |
| **Temperature shift** | 34°C (Day 4) |
| **DO set point** | 40% |
| **pH set point** | 6.90 ± 0.20 |
| **Medium** | CD OptiCHO + 6 mM L-Glutamine + 0.25 g/L Cell Boost 4 |
|  |  |
| **Feed type** | Cell Boost 7a and 7b |
| **Supplement addition** | L-glutamine: 4 mM on Day 2  250 g/L Glucose* as needed to maintain ≥3.5 g/L (post-feed target) |
| **Harvest criteria** | Day 15 or ≤ 50% viability |
