# Supplementary material for: cGMP production and analysis of BG505 SOSIP.664, an extensively glycosylated, trimeric HIV‐1 envelope glycoprotein vaccine candidate
Source: Biotechnol Bioeng. 2017 Dec 11;115(4):885–99. doi: 10.1002/bit.26498 (PMC5852640; doi:10.1002/bit.26498)
Supplement: Supplementary file 3 — Table S2. Table S2. Amino acid analysis [file BIT-115-885-s003.docx]

**Table S2.**

| Method | Result |
| --- | --- |
| Amino Acid Analysis | **Mol % Comparison (detected/theoretical)** |
|  | Aspartate: 109.5% |
|  | Glutamate: 116.1% |
|  | Serine: 97.2% |
|  | Histidine: 99.3% |
|  | Glycine: 103.2% |
|  | Threonine: 99.9% |
|  | Arginine: 123.7% |
|  | Alanine: 116.6% |
|  | Tyrosine: 85.7% |
|  | Cysteine: 1.8% |
|  | Valine: 90.1% |
|  | Methionine: 55.0% |
|  | Phenylalanine: 105.3% |
|  | Isoleucine: 86.1% |
|  | Leucine: 102.7% |
|  | Lysine: 102.4% |
|  | Proline: 112.4% |
|  | Average: 94.5% ^2^  Average: 103.3% ^3^ |
