# Supplementary material for: cGMP production and analysis of BG505 SOSIP.664, an extensively glycosylated, trimeric HIV‐1 envelope glycoprotein vaccine candidate
Source: Biotechnol Bioeng. 2017 Dec 11;115(4):885–99. doi: 10.1002/bit.26498 (PMC5852640; doi:10.1002/bit.26498)
Supplement: Supplementary file 5 — Table S4. Excipient screening study [file BIT-115-885-s005.docx]

**Table S4.**

| **Buffer** | **pH** | **NaCl (mM)** | **Excipient** | **Storage Temperature** |
| --- | --- | --- | --- | --- |
| Sodium citrate  (10 mM)  pKa = 6.4 | 6.5 | 100 | -- | 5 ± 3°C |
|  |  |  | -- | 25 ± 2°C at  60 ± 5% RH |
|  |  |  | 150 mM Arginine |  |
|  |  |  | 250 mM Sucrose |  |
|  | 7.0 | 100 | -- | 5 ± 3°C |
|  |  |  | -- | 25 ± 2°C at  60 ± 5% RH |
|  |  |  | 150 mM Arginine |  |
|  |  |  | 250 mM Sucrose |  |
| Sodium phosphate  (20 mM)  pKa = 7.2 | 7.5 | 100 | -- | 5 ± 3°C |
|  |  |  | -- | 25 ± 2°C at  60 ± 5% RH |
|  |  |  | 150 mM Arginine |  |
|  |  |  | 250 mM Sucrose |  |
|  | 8.0 | 100 | -- | 5 ± 3°C |
|  |  |  | -- | 25 ± 2°C at  60 ± 5% RH |
|  |  |  | 150 mM Arginine |  |
|  |  |  | 250 mM Sucrose |  |
| Tris  (20 mM)  pKa = 8.1 | 7.5 | 100 | -- | 5 ± 3°C |
|  |  |  | -- | 25 ± 2°C at  60 ± 5% RH |
